# Insights of health district managers on the implementation of primary health care outreach teams in Johannesburg, South Africa: a descriptive study with focus group discussions

**DOI:** 10.1186/s12960-017-0183-6

**Published:** 2017-01-21

**Authors:** Shabir Moosa, Anselme Derese, Wim Peersman

**Affiliations:** 1 Department of Family Medicine, University of Witwatersrand, Johannesburg, South Africa; 2 Department of Family Medicine and Primary Health Care, Ghent University, Ghent, Belgium; 3 Knowledge Centre Brussels Integrated Care, Erasmus University College Brussels, Brussels, Belgium

**Keywords:** Primary health care, Community healthcare workers, Outreach teams, Africa, Human resources, Policy

## Abstract

**Background:**

Primary health care (PHC) outreach teams are part of a policy of PHC re-engineering in South Africa. It attempts to move the deployment of community health workers (CHWs) from vertical programmes into an integrated generalised team-based approach to care for defined populations in municipal wards. There has little evaluation of PHC outreach teams. Managers’ insights are anecdotal.

**Methods:**

This is descriptive qualitative study with focus group discussions with health district managers of Johannesburg, the largest city in South Africa. This was conducted in a sequence of three meetings with questions around implementation, human resources, and integrated PHC teamwork. There was a thematic content analysis of validated transcripts using the framework method.

**Results:**

There were two major themes: leadership-management challenges and human resource challenges. Whilst there was some positive sentiment, leadership-management challenges loomed large: poor leadership and planning with an under-resourced centralised approach, poor communications both within the service and with community, concerns with its impact on current services and resistance to change, and poor integration, both with other streams of PHC re-engineering and current district programmes. Discussion by managers on human resources was mostly on the plight of CHWs and calls for formalisation of CHWs functioning and training and nurse challenges with inappropriate planning and deployment of the team structure, with brief mention of the extended team.

**Conclusions:**

Whilst there is positive sentiment towards intent of the PHC outreach team, programme managers in Johannesburg were critical of management of the programme in their health district. Whilst the objective of PHC reform is people-centred health care, its implementation struggles with a centralising tendency amongst managers in the health service in South Africa. Managers in Johannesburg advocated for decentralisation. The implementation of PHC outreach teams is also limited by difficulties with formalisation and training of CHWs and appropriate task shifting to nurses. Change management is required to create true integrate PHC teamwork. Policy review requires addressing these issues.

## Background

There is a growing embrace of community health workers (CHWs) and task shifting to junior professionals as a response to human resource shortages in primary health care. A key difficulty is how to integrate them into health systems to provide comprehensive people-centred primary health care [1–3]. The South African government has been striving to deliver improved public primary health care service for all since 1994, with infrastructural initiatives based on a new district health system. However services remain fragmented, with most doctors and nurses located in the private sector, resulting in poor health outcomes [4]. This is a function of the apartheid past as well as failures in current health leadership-management in South Africa [5].

National Health Insurance (NHI) in South Africa is an attempt to address this public-private inequity, mostly by funding changes [5, 6]. NHI includes service delivery reform, termed primary health care (PHC) re-engineering, as a shift towards more prevention. There are three streams in PHC re-engineering: municipal ward-based PHC outreach teams, school health teams, and district-based clinical specialist teams (to support maternal and child health outcomes mostly) [4, 6]. CHWs have been described by government officials as poorly coordinated, inadequately trained and supervised, randomly distributed in verticalised programmes and struggling with links between the community and fixed clinics [7]. PHC outreach teams are an attempt to change CHWs deployment to a more integrated, team-based approach responsible for defined populations and strengthening interactions between services and service users [8].

The PHC outreach team consists of a professional nurse (a senior nurse trained over three years), supported by a health promoter and environmental health officer, leading a team of six CHWs within the geographic area of a municipal ward, as the unit of election of councillors to local government. Each CHW takes care of 250 families. The PHC outreach team is supposed to work with another professional nurse and an enrolled nurse (a junior nurse trained over 2 years) at the clinic to provide comprehensive care to this population, from health promotion to treatment of minor ailments [8]. CHWs are to have a standardised scope of work; clearly defined roles, responsibilities and job description; certified training; specified qualification requirements; employment mechanisms; training and supervision packages; and remuneration and condition of service [4]. The CHWs do mostly household profiling, screening, and health education, with supervision by their professional nurse team leader. The CHWs refer problem patients to their supervising professional nurse and/or the clinic nurses and then do community-based follow-up of these patients with health education and home-based care. There may be more than one team per ward, depending on the population.

Johannesburg is one of five health districts/municipal districts in Gauteng Province, which is one of the nine provinces in South Africa. Johannesburg is home to 4.4 million people [9]. Most smaller clinics are managed by local government/municipal managers (known as the City of Johannesburg (CoJ)), whilst the fewer larger community health centres (CHC) are managed by managers appointed by provincial government for the health district (known as Johannesburg Metro). Johannesburg Metro is the principal manager of the health district and CoJ is deemed an agent, in terms of the National Health Act of 2003. Each has a set of programme managers overseeing all verticalised services, e.g. non-communicable diseases or HIV-TB for Johannesburg. There had been various efforts in Johannesburg since 2009 on developing community-oriented primary care (COPC); however, there has been little public examination of the challenges in implementing PHC Outreach Teams, as a stream of PHC re-engineering.

The aim of this study was to understand the views of district health managers in Johannesburg on the implementation of PHC outreach teams.

## Methods

### Study design

This was a qualitative study, using focus group discussions with managers from both the City of Johannesburg and Johannesburg Metro. Invitations were sent to all senior managers from both local and provincial government in the Johannesburg Health District. These senior managers were at district director, deputy district director and assistant director level and included sub-district managers, programme managers (including PHC Outreach Team managers), senior doctors and specialists.

### Data collection

Three meetings were organised over a period of 2 months, October to November 2013, where managers responded to particular questions and built on these responses in the subsequent meetings. Written informed consent was obtained from each participant before a 45–60-min focus group discussion was conducted following a standard operating procedure and discussion guide. A total of nine focus group discussions were conducted over the three meetings. Trained researchers facilitated the meetings and discussions. All discussions were conducted in English and digitally recorded.

The first meeting was used to brief all participants on the policy and then addressed the following issue:

1. What are your views on the implementation of the PHC outreach programme across your health district?

In the second meeting, after feedback and reflection from the first meeting, the following question was addressed:

2. What are your views on human resource in implementation of the PHC outreach programme across your health district?

The third meeting reviewed the previous discussions and proceeded to focus on the following two questions:

3. What are your views on integrated PHC teamwork in the implementation of the PHC outreach programme across your health district?
4. What are your views on the challenges of ethics, process and power in the implementation of the PHC outreach programme across your health district?

### Data analysis

The audio recordings were transcribed verbatim. Qualitative data analysis followed the framework method [10]. After the research team had familiarised themselves with the data, and a coding index was agreed upon, all transcripts were systematically coded using NVivo9. The lead researcher (SM) supervised the process. The research team collectively analysed the anonymised coded material in order to develop key findings.

### Ethical approval

The University of the Witwatersrand’s Human Research Ethics Committee (Medical) (M130116) gave ethical approval in February 2013. Participants were not offered any monetary reward for participating in the study. The data produced in the project remain confidential, and the participants remain anonymous in all transcripts and analyses. Approval to conduct the study was also given by Gauteng Provincial Department of Health and by the Johannesburg Health District acting Chief Director.

## Results

There were four groups of participants: senior and junior managers from local government (LG) (City of Johannesburg) and provincial government (PG) (Johannesburg Metro). Some senior district managers had deployed junior sub-district managers to represent them (Table 1). There were three meetings (with number of focus groups formed in brackets): 1 October 2013 (4), 23 October 2013 (3) and 13 November 2013 (2).

**Table 1:** Distribution of managers participating at the different focus group discussions

	Participants	Attended 1 October 2013	Attended 23 October 2013	Attended 13 November 2013
Senior managers (PG)	15	11	7	9
Senior managers (LG)	5	5	3	3
Junior managers (PG)	10	9	2	5
Junior managers (LG)	11	8	2	3
Total attended	41	33	14	20

PG provincial government

LG local government

The results are presented under two main sections: leadership-management challenges and human resource challenges (Table 2). Quotes given by respondents are labelled according to the meeting and focus group number, e.g. 3.2 is focus group two in meeting three.

**Table 2:** Themes and sub-themes

Themes	Sub-themes
A) Leadership and management challenges	Positive experiences Poor leadership and planning Poor communications and consultation Concerns with impact on services Poor integration
B) Human resource challenges	Community health workers Nurses Extended team

### Leadership and management challenges

Whilst the programme was seen as positive, this sentiment was overwhelmed by a number of leadership and management challenges: poor leadership and planning, poor communications and consultations, concerns with impact on current services and poor integration.

#### Positive experiences

Participants felt that the programme improved access and, more importantly, reduced the burden on clinics. It was reminiscent of older times with community nursing, destroyed due to the post-apartheid focus of nursing on curative care:

It is more a preventative and promotive approach…rather than the curative approach we have in the past … look at the whole ARV (Anti-Retro Viral) programme that is really twisting the way that we use our resource (FGD 1.4)

#### Poor leadership and planning

Participants were uncomfortable with different approaches and wanted a standardised approach. Participants ambivalently also felt that this was an opportunity to see what works:

In Jo’burg, there were quite a lot of different views… Everyone has his (own) concept and goes everywhere (FGD 1.2)

If it works well in this clinic let them do it that way (FGD 3.2)

They felt that leadership was poor and uninformed, with limited capacity in the district management responsible for PHC outreach to manage this large project. Participants shared that planning had been poor for years. They thought that the policy needed a clearly phased implementation plan that accounted for communities and contexts:

If you look at the team at district level, it is also very thin. This is a very big district (FGD 1.2)

They did not approve of the current centralised planning and recommended that planning become decentralised and devolved. They said that those on the ground, especially facility managers (as opposed to sub-district managers), could better implement the programme:

Is it a top down approach or (should it not be) a bottom up approach? … And that is why I am saying, that we need to have a proper plan. (FGD 1.2)

They thought that the programme needed to be adequately resourced with a sustainable budget. There were serious challenges with medical equipment, materials and space for CHWs. This programme was seen as a way to save money in the long term:

You all talk about PHC re-engineering, but where is the financial re-engineering? … “here is the money for, that is ring-fenced to kick start this sort of process”, and slowly merge it into the normal budgetary processes (FGD 1.1)

#### Poor communications and consultations

It was also felt that CHWs and staff were not orientated to what each were doing, missing an opportunity for integration and creating an ethical challenge. It was suggested that this influx would decrease over time as prevention and promotion efforts improve. CHWs were seen now as first contact within the health system, doing more than just referring to the clinic, as they appear to be currently doing. They suggested that PHC re-engineering required a change to management at a facility level, ensuring that everyone understands this process.

This lack of communication and consultation was not only amongst managers but also between the teams and their communities. Participants intimated that there was poor communication and advocacy around this programme, including with councillors and the community, as people were hearing about the programme in the corridors. There was a clear need for everyone to be properly orientated to the programme:

It was not properly communicated. If I may tell you, I’m confused right now (FGD 1.1)

Managers felt that the programme was not well informed of power relations between councillors, politically linked non-government organisations, clinic committees appointed by provincial government and ward committees appointed by local government:

It’s more of political than anything else because you’ll find that (the NGO responsible for CHWs) is somebody who’s linked to so and so and he has been given a tender (FGD 3.2)

Participants debated whether this programme was invading the privacy of people in their homes and imposing on people’s autonomy:

Let’s look at the issue of invading people’s privacy…. Do we have right to do that? Like health now is imposing on people, people are no longer having decisions to make. (FGD 3.1)

#### Concerns with impact on current services

There were major concerns about the influx of patients on an already overburdened service, with high rates of community movement. Clinic staff sees this project as extra work, causing conflict with team leaders. There were concerns about the existing culture of pushing queues:

There is chaos in one of the facilities because people take this as an extra to what they are doing (FGD 2.1)

I’ve got this mentality of saying: I’ve done my forty (patients); she must do her forty (patients) (FGD 2.2)

Participants said that encouraging patients to visit an overworked staff at a clinic offering poor standard care was unethical:

We talk about reengineering, can we reengineer a system that is broken or problematic. Do you see ethical concerns with that maybe? (FGD 3.2)

#### Poor integration

The participants shared that this programme is being created as another verticalised, silo programme, with poor integration even between the three streams of PHC Re-engineering. They were also concerned regarding integration with other vertical programmes in the district, with poor briefing of programme managers and social welfare, and the divide between local and provincial government. The programme should belong to everyone in the district with the whole system re-arranged around a common goal:

I want to say that this must not be seen as a separate, different programme altogether. It’s part of re-engineering primary healthcare (FGD 2.2)

This whole model or process is huge … we are running two parallel services in one area. Local and provincial, and there is nowhere where they come together (FGD 3.1)

### Human resource challenges

Human resources were seen as a serious challenge: community health workers (CHWs), nurses and the extended team:

#### Community health workers

Two key CHWs concerns were addressed: formalising the functioning of CHWs and training CHWs. Participants were concerned about the fragmented distribution and unclear roles of CHWs. They felt that CHWs were doing outstanding work and supported generalist CHWs:

We are getting a whole lot of different people and … duplicating processes … what they are doing is really outstanding in our community (FGD 1.1)

They were concerned about conditions of CHWs as non-employees: low stipend, non-payment, limited capacity and high burden. They wanted a planned strategy for CHWs, including career progression and professional regulation. They were concerned about security risk, space and logistical support. Participants felt that CHWs need to be selected based on some criteria, more than a matriculation:

Please let’s employ them, be part of the staff establishment (FGD 3.1)

Participants were concerned about CHWs training considering their responsibilities:

What is a ten-day course going to do? Nothing (FGD 3.1)

Participants felt that programme managers should be included in a comprehensive and standardised training plan. There was a concern that the reliance on team leaders to provide ongoing training for CHWs was misplaced.

Whilst managers felt that the community viewed CHWs as professional and favoured CHWs being developed as professionals, they questioned CHWs as professionals, largely with their limited training:

We are sending poorly trained people into communities to do a bad job, that’s an ethical problem (FGD 3.2)

There was a tension between the views that CHWs coming from their communities would improve their familiarity and work and the counter view that this closeness made communities uncomfortable about confidentiality. There were concerns about CHWs safety, identification, debriefing, risk for contracting diseases and professional accountability:

We as professionals, have nursing council, etc. Who covers them (for professional accountability)? (FGD 3.2)

#### Nurses

Managers considered the nursing shortage a serious challenge. It was felt that wards were big and needed more than one team. This could not come from current nursing staff:

At the moment there is such a shortage of staff to implement this, it really becomes a serious challenge (FGD 2.2)

Managers were using school health nurses and district health nurses as team leaders, as they were already working outside the clinic. These nurses were expected to manage this in addition to their current roles, resulting in unhappiness. Clinic staff did not feel responsible for these patients referred from CHWs as they have a team leader. This was especially with a culture of quotas, long tea times and resistance to change:

I’ve done my forty patients. If you, yours were difficult one, you are still at number twenty-eight, she was not going to help you. To her, even if it’s two o’clock, I’m off; I’m having my long tea. (We need) to break that mentality, that everybody owns everybody else (FGD 2.2)

Managers felt that the team leader should be a dedicated PHC nurse (a professional nurse with post-graduate 18 months training as clinician) so that patients were seen in the community and not referred to other nurse clinicians. Others felt that dedicated clinicians at the clinic could see patients referred by CHWs. It was felt that the clinic itself needed to take responsibility for all patients, especially with nurse shortages. Managers were wary of poorly trained new nurses:

My view is (that the team leader) should be someone who is a clinician (FGD 2.2)

A nurse just out of college shouldn’t be taken. They should have some experience (FGD 2.1)

#### Extended team

There was also mention of others outside the basic team of nurse team leader and community health worker:

I think there would be a basic team, and their support team and what do you call it, extended team? Not sure. (FGD 3.2)

Whilst doctors were seen as useful to the PHC outreach programme, these managers viewed it as impractical with doctor shortages. The role of the doctor was seen as just providing clinical curative services to those patients referred:

In the ward we could have one doctor for three or four wards, okay (pause) (so) let’s not get carried away. (FGD 1.4)

The social worker was felt necessary. Managers mentioned the role of clinical associate, social worker, health promoters, environmental health officers and clinical psychologists.

## Discussion

It is significant that managers in Johannesburg see PHC re-engineering, with its preventive approach and re-orientation from specialised to generalist CHWs, as a step in the right direction. It underlines government intent towards a more integrated horizontal approach [7] and its value in PHC [11].

However, managers in Johannesburg quickly pointed out implementation challenges of leadership-management, citing poor planning, poor integration and poor communication. Middle managers suggested that PHC outreach teams be central to the district’s re-arrangement and functioning. They asked the question: ‘we talk about re-engineering, can we re-engineer a system that is broken?’ and questioned the ethics of overwhelming service delivery. Kautzky and Tollman [12] feel that there needs to be an intense effort to salvage the currently over-bureaucratized and rigid primary care service. PHC reform needs a redefinition of strategic and organisational planning of the district health system in South Africa [13]; otherwise, patients will continue to get lost in the system because of lack of integration, as government points out [8]. Conceptualizations of integration appear poor. A salient view is that people-centredness should be the organising focus of integrated PHC [14]. However, the tendency is for managers, especially senior district managers, to organise and integrate around themselves and the bureaucratic structures they create.

This was evident with managers cautioning about the impact on current services with the current culture of ‘pushing queues’. They thought that clinic staff would see this as extra work and resist changes. Sub-district managers say that facility and operational managers are unable to see the big picture [15]. The shortages of equipment, supplies and transport seemed to reflect a currently dysfunctional service that needed ‘financial re-engineering’ rather than a lack of resources. South Africa already spends the second highest percentage of government expenditure on health in Africa [16]. These challenges of lack of leadership, integration and service take up in PHC Outreach teams are not unique to Johannesburg [17].

Current command and control approaches are seen as flawed with a bottom-up approach suggested as critical [18]. Managers in Johannesburg recommended that management and planning should be decentralised to facility managers. Decision space is required for managers [19]. A service interface empowered as close to the community is likely to be patient-, person- and people-centred [12]. Decentralisation is an important call in Africa [20, 21] and South Africa [22]. There is a need for a complex adaptive systems thinking approach. There needs to pro-active management, local service improvement priorities and population accountability [23]. Managerialism in the public service can demobilise communities, rendering community participation very patronising [24]. Familiarity with the community by all providers improves community engagement and service integration [25] but also risked the invasion of privacy and confidentiality. Managers shared this sentiment. They were also worried about patient autonomy with the PHC outreach teams.

Managers considered human resource challenges as a serious challenge, with most of their focus on CHWs. They saw the service profoundly shifting to make the CHWs the first point of contact with the formal health system and addressing the current verticalised programmatic fragmentation of care. This seems aligned with national intent [8, 26] and good practice internationally [27–29]. CHWs understand contexts and social situations from which their patients come and manage a myriad of social challenges within families and communities, congruent with international evidence of promising benefits [30].

Managers’ in Johannesburg wanted the formalisation of CHWs, especially in respect of incentives. This is an important consideration in a systematic review of design factors influencing performance of CHWs in low- and middle-income countries [1]. Managers felt that the CHW workload was very heavy and their working conditions difficult, citing the lack of space, stationery and equipment. This is not unique to South Africa [31] and influences CHWs productivity [32]. CHWs deployment requires a strengthening of the existing health system and an enabling environment for CHWs [29, 33]. Managers shared sentiments in line with international calls for better recruitment, standardisation and performance management [33, 34].

Managers felt the need for more formalised CHW training, with the amount of complexity being thrust on CHWs without the ability to cope. This concern is evident in other programmes in Africa [35], with community criticism of CHWs competence jeopardising the programme [36]. Managers in Johannesburg felt that the training focus should be local and more than just a ten-day course.

CHWs are politically powerful being ‘agents of the state’ being very present in the community and potentially holding the key to access of health services. On the other hand, their employment may render them as bureaucratic extensions of a dysfunctional health service, where their role as advocates for social change is replaced by a predominantly technical community management function [37, 38]. Politicians see CHWs as a panacea to all their problems and there is a danger that the CHW programme will take on more than it can deliver [26, 29].

Formalising CHWs would require significant budget but costs could be modest [27, 39]. Managers questioned the ethics of holding CHWs in a state of precarity: exposed to risks, poorly supported, poorly and often irregularly paid. This lack of care for the carer is symptomatic of the entire public service [40] and creates the impression that CHWs are readily disposable.

Government has premised the PHC outreach team on professional nurses as team leaders [17]. Participants saw this requirement as unrealistic and suggested using enrolled nurses instead. Managers in Johannesburg questioned the use of school and district nurses and the choice of inexperienced nurses as new team leaders. Managers suggested that teams be linked to a PHC Nurse based at the clinic, seeing referred patients and able to see patients comprehensively. However, such teamwork is confounded by their concern about the culture of quotas and long teas amongst staff. A major reason for dissatisfaction with health services is the non-responsiveness of the nursing profession and the non-caring attitude of health care personnel [18, 41]. Change management, dealing with resistance, appears a key hurdle on which implementation of PHC Re-engineering appears to be stumbling [42, 43]. Task shifting can be useful but overburdening lowly-paid health workers with very complex tasks can be counterproductive, whatever the short-term cost benefits may appear. Integration with task shifting needs local clinical leadership to manage the staff-skills mix [44]. Task shifting is more than substitution and delegation. It includes supervision, enhancement, mentoring and innovation [45]. Managers in Johannesburg felt that the capacity of facility managers was limited and needed changing. Clinic managers are struggling with the same problems as general staff: poor practice environment, workload, professional support, training, pay, standards of care and security [46]. Whether leadership training is enough to address the current culture is moot. The relegation by managers of doctors to clinical curative work and the poor use of clinical associates is of concern [47–50]

There are limitations to this study. It obtains views of only one health district in Gauteng. Some key members of management are noted as missing. The research team involvement in previous work on PHC outreach teams may have biased results. Further such research is required in other districts. The study was done in 2013 and may be dated.

## Conclusions

There needs to be a review of the South African policy of PHC re-engineering, especially related to district management, roles and norms of staffing, formalisation of CHWs and their training, especially as health professionals in line with various proposals [51–53]. Implementation of PHC outreach teams needs to include a true re-engineering of PHC at a health district level. Implementation should be allied with stronger devolution of power to facility level, together with appropriate resources. Communities should be actively engaged in the process.

## Abbreviations

CHW: Community health worker
CoJ: City of Johannesburg
NHI: National Health Insurance
PHC: Primary Health Care
